# Differential effectiveness of tyrosine kinase inhibitors in 2D/3D culture according to cell differentiation, p53 status and mitochondrial respiration in liver cancer cells

**DOI:** 10.1038/s41419-020-2558-1

**Published:** 2020-05-07

**Authors:** María A. Rodríguez-Hernández, Raquel Chapresto-Garzón, Miryam Cadenas, Elena Navarro-Villarán, María Negrete, Miguel A. Gómez-Bravo, Victor M. Victor, Francisco J. Padillo, Jordi Muntané

**Affiliations:** 1 Institute of Biomedicine of Seville (IBiS), Hospital University “Virgen del Rocío”/CSIC/University of Seville, Seville, Spain; 2 Spanish Network for Biomedical Research in Hepatic and Digestive diseases (CIBERehd), Institute of Health Carlos III (ISCIII), Madrid, Spain; 3 Department of General Surgery, Hospital University “Virgen del Rocío”/CSIC/University of Seville/IBIS, Seville, Spain; 4 Service of Endocrinology, University Hospital Doctor Peset, Foundation for the Promotion of Health and Biomedical Research in the Valencian Region (FISABIO), Valencia, Spain; 5 Department of Physiology, University of Valencia, Valencia, Spain

**Keywords:** Predictive markers, Liver cancer

## Abstract

Sorafenib and Regorafenib are the recommended first- and second-line therapies in patients with advanced hepatocellular carcinoma (HCC). Lenvatinib and Cabozantinib have shown non-inferior antitumoral activities compared with the corresponding recommended therapies. The clinical trials have established recommended doses for each treatment that lead different blood concentrations in patients for Sorafenib (10 µM), Regorafenib (1 µM), Lenvatinib (0.1 µM), and Cabozantinib (1 µM). However, very low response rates are observed in patients attributed to intrinsic resistances or upregulation of survival signaling. The aim of the study was the comparative dose–response analysis of the drugs (0–100 µM) in well-differentiated (HepG2, Hep3B, and Huh7), moderately (SNU423), and poorly (SNU449) differentiated liver cancer cells in 2D/3D cultures. Cells harbors wild-type p53 (HepG2), non-sense p53 mutation (Hep3B), inframe p53 gene deletion (SNU423), and p53 point mutation (Huh7 and SNU449). The administration of regular used in vitro dose (10 µM) in 3D and 2D cultures, as well as the dose–response analysis in 2D cultures showed Sorafenib and Regorafenib were increasingly effective in reducing cell proliferation, and inducing apoptosis in well-differentiated and expressing wild-type p53 in HCC cells. Lenvatinib and Cabozantinib were particularly effective in moderately to poorly differentiated cells with mutated or lacking p53 that have lower basal oxygen consumption rate (OCR), ATP, and maximal respiration capacity than observed in differentiated HCC cells. Sorafenib and Regorafenib downregulated, and Lenvatinib and Cabozantinib upregulated epidermal growth factor receptor (EGFR) and mesenchymal–epithelial transition factor receptor (c-Met) in HepG2 cells. Conclusions: Sorafenib and Regorafenib were especially active in well-differentiated cells, with wild-type p53 and increased mitochondrial respiration. By contrast, Lenvatinib and Cabozantinib appeared more effective in moderately to poorly differentiated cells with mutated p53 and low mitochondrial respiration. The development of strategies that allow us to deliver increased doses in tumors might potentially enhance the effectiveness of the treatments.

## Introduction

Liver cancer is the sixth most common cancer and the fourth most frequent cause of cancer-related death worldwide^1^. Sorafenib is the standard of care for patients in advanced hepatocellular carcinoma (HCC) stage^2,3^. Different phase III clinical trials showed Lenvatinib was statistically non-inferior to Sorafenib in overall median survival as first-line therapy^4^, and Regorafenib was recommended as second-line therapy in patients nonresponsive but tolerant to Sorafenib^5^. Cabozantinib has also showed positive results as a second-line treatment for advanced HCC^6^.

Sorafenib is a multityrosine kinase inhibitor, and Raf-mitogen-activated protein kinase (MAPK)–extracellular signal-regulated kinase (ERK) signaling^7^, with potent antiproliferative and proapoptotic properties in liver cancer cells^8,9^. The induction of cell death by Sorafenib has been related to a rise in PUMA and BIM, as well as a reduction in induced myeloid leukemia cell differentiation protein (Mcl-1) and survivin^9^. Sorafenib (10 µM) induced a sustained endoplasmic reticulum stress promoting a rise of Bim_EL_ expression that mediates the shift from survival autophagic pathway to apoptosis in HepG2^10^. Regorafenib, Lenvatinib, and Cabozantinib have also been shown to promote cell death and antiproliferative properties in vitro and in vivo, using different cancer cells lines^11–13^.

The aim of the present study was to determine the impact of cell differentiation stage and p53 genetic status in the effectiveness of tyrosine kinase receptor inhibitors (TKIs) in liver cancer cells. A comparative dose–response analysis of the antiproliferative and proapoptotic effectiveness of Sorafenib, Regorafenib, Lenvatinib, and Cabozantinib was carried out in 2D and 3D cultured liver cancer cells. The study includes HCC cell lines in well- (HepG2, Hep3B, and Huh7), moderately (SNU423), and poorly (SNU449) differentiated stages, as well as expressing wild-type p53 (HepG2), non-sense p53 mutation (Hep3B), inframe p53 gene deletion (SNU423), and p53 point mutation (Huh7 and SNU449)^14,15^.

## Materials and methods

### Drugs

Sorafenib (FS10808), Regorafenib (FR16116), Cabozantinib (FD59688), and Lenvatinib (FC75063) were obtained from Carbosynth Limited (Berkshire, UK). Drugs were solved in dimethyl sulfoxide as stock solution (100 mM).

### Primary human hepatocytes, cell lines, and culture conditions

Human hepatocytes were prepared from liver biopsies obtained from two patients (one female and one man, aged 43 ± 6.0 years) submitted to surgical resection for liver tumors after obtaining patients’ written consent. The study protocol was approved by the Ethical Committee of the Institution. The isolation of human hepatocytes was based on the two-step collagenase procedure, and cells were cultured as previously described^16^. HepG2 (HB-8065™, American Type Culture Collection (ATCC)/LGC Standards, SLU, Barcelona, Spain), Hep3B (HB-8064™, ATCC/LGC Standards), Huh7 (Apath LLC, Brooklyn, USA), SNU423 (CRL-2238, ATCC/LGC Standards), and SNU449 (CRL-2234, ATCC/LGC Standards) cell lines were cultured in supplemented Minimum Essential Medium with Earle’s Balanced Salts (GE Healthcare HyClone, Boston, USA) at 37°C in a humidified incubator with 5% CO_2_.

Multicellular spheroids were generated according to the liquid overlay technique (Supplementary Fig. 1)^17^. The spheroid area (µm^2^), non-trypan and trypan blue-stained cells, Ki67- and TUNEL-positive cells, and caspase-3 activity were analyzed in spheroids (%, fold over control). Cells were also cultured in monolayer (100,000 cells/cm^2^). Treatments (0–100 µM) were administrated 24 h after plating, and cell lysates obtained at different time points for measuring cell proliferation and apoptosis as previously described^10^.

### Cell proliferation

Cell proliferation was assessed in 3D and 2D cultured cells. Non-trypan blue-stained viable cells were quantified in trypsin-treated spheroids, as well as Ki67-positive cells were determined in 4% paraformaldehyde-fixed spheroid sections (5 µ) by immunocytochemistry as described previously^18^. Immunofluorescence analysis was performed using Olympus BX61 microscope. Fluorescence quantification was performed using Leica Application Suite Advanced Fluorescence software and ImageJ software. Cell proliferation rate was also measured by bromodeoxyuridine (BrdU) incorporation in 2D cultured cells using the recommendation included in the commercial assay (11647229001, Roche Diagnostics, Basel, Switzerland)^18^. Data are shown as the relative absorbance at 370 nm, using as reference wavelength 492 nm (Absorbance, %, fold over control) using an Infinite 200 PRO Microplate Reader (TECAN, Männedorf, Switzerland).

### Measurement of cell death

Cell death was assessed either in trypsin-isolated cells with the measurement of trypan blue-stained non-viable cells in spheroids, or TUNEL-positive cells in 4% paraformaldehyde-fixed spheroid sections (5 µ) useful for DeadEnd™ Fluorometric TUNEL System (G3250, Promega, Madison, Wisconsin, USA). Caspase-3-associated activity was determined using Caspase-Glo® Assay Systems (G8091, Promega) in 3D and 2D cultured cells. Both, spheroids and cells were lysed as described above and treated with Caspase-Glo® Reagent in an “add-mix-measure” format, resulting in caspase-3-dependent cleavage of the substrate and generation of a “glow-type” luminescent signal. The signal generated is proportional to the amount of caspase activity. The values were extrapolated into a calibration curve included in the assay and the results are shown as the relative light units (RLU, %, fold over control).

### Mitochondrial respiration

Mitochondrial respiration in intact adhered cells was assessed with the XFp Cell Mito Stress Test Kit in a XFp Extracellular Flux Analyzer (Seahorse Bioscience, Billerica, Massachusetts, USA). Oxygen consumption rate (OCR) was measured in cells 48 h after plating and data normalized to total protein content per well (pmol/min/µg protein) as previously described^19^.

### Expression of tyrosine kinase receptors

The expression of epidermal growth factor receptor (EGFR), vascular endothelial growth factor receptor (VEGFR), platelet-derived growth factor receptor (PDGFR), fibroblast growth factor receptor (FGFR), and mesenchymal–epithelial transition factor receptor (c-Met) was determined by SDS–PAGE electrophoresis coupled to western blot analysis. Proteins (50–100 μg) were separated by Any kD™ Criterion™ TGX Stain-Free™ Protein Gel, 18 well, 30 µl (#5678124, BioRad, Hercules, California, USA), and transferred to PVDF membranes. The system uses stain-free technology, which is a method that appears to be more reliable as a protein loading control than the measurement of housekeeping proteins^20^. The membranes were incubated with the corresponding commercial primary EGFR (#4267 S), VEGFR (#2479 S), PDGFR (#3174 S), FGFR (#3472 S), and c-Met (#8198 S) obtained from Cell Signaling Technology (Danvers, Massachusetts, USA), and secondary anti-rabbit (sc-2004) IgG-HRP-labeled (Santa Cruz Biotechnology, Inc, Dallas, Texas, USA) antibodies revealing protein content by Clarity™ Western ECL substrate (170–5061, BioRad) and analyzed in a ChemiDoc™ Touch Imaging System. The densitometric analysis (Densitometry, %, fold over control) was performed using the software Image Lab 6.0 of BioRad.

### Statistical analysis

All results are expressed as mean ± SD of independent experiments (*n* = 2–8). Data were compared using the analysis of variance with the Least Significant Difference’s test as post hoc multiple comparison analysis (homogeneity of variances) or Games-Howell (non-homogeneity of variances). If Shapiro–Wilks’s test showed non-normal distribution of data nonparametric Kruskal–Wallis coupled to Mann–Whitney *U* post hoc analysis with Finner’s correction was done. The level of significance was set at *p ≤ 0.05, **p ≤ 0.01, and ***p ≤ 0.001 between groups. The groups with statistically significant differences (*p* ≤ 0.05) were also indicated with different letters. The sample size was determined using Granmo v7 software. All statistical analyses were performed using the IBM SPSS Statistics 19.0.0 (SPSS Inc., IBM, Armonk, New York, USA) software.

## Results

### Differential antiproliferative and proapoptotic properties of Sorafenib, Regorafenib, Lenvatinib, and Cabozantinib administered at a regular used in vitro dose (10 µM) in 3D and 2D cultured-differentiated HCC with different p53 status

**Fig. 1:**
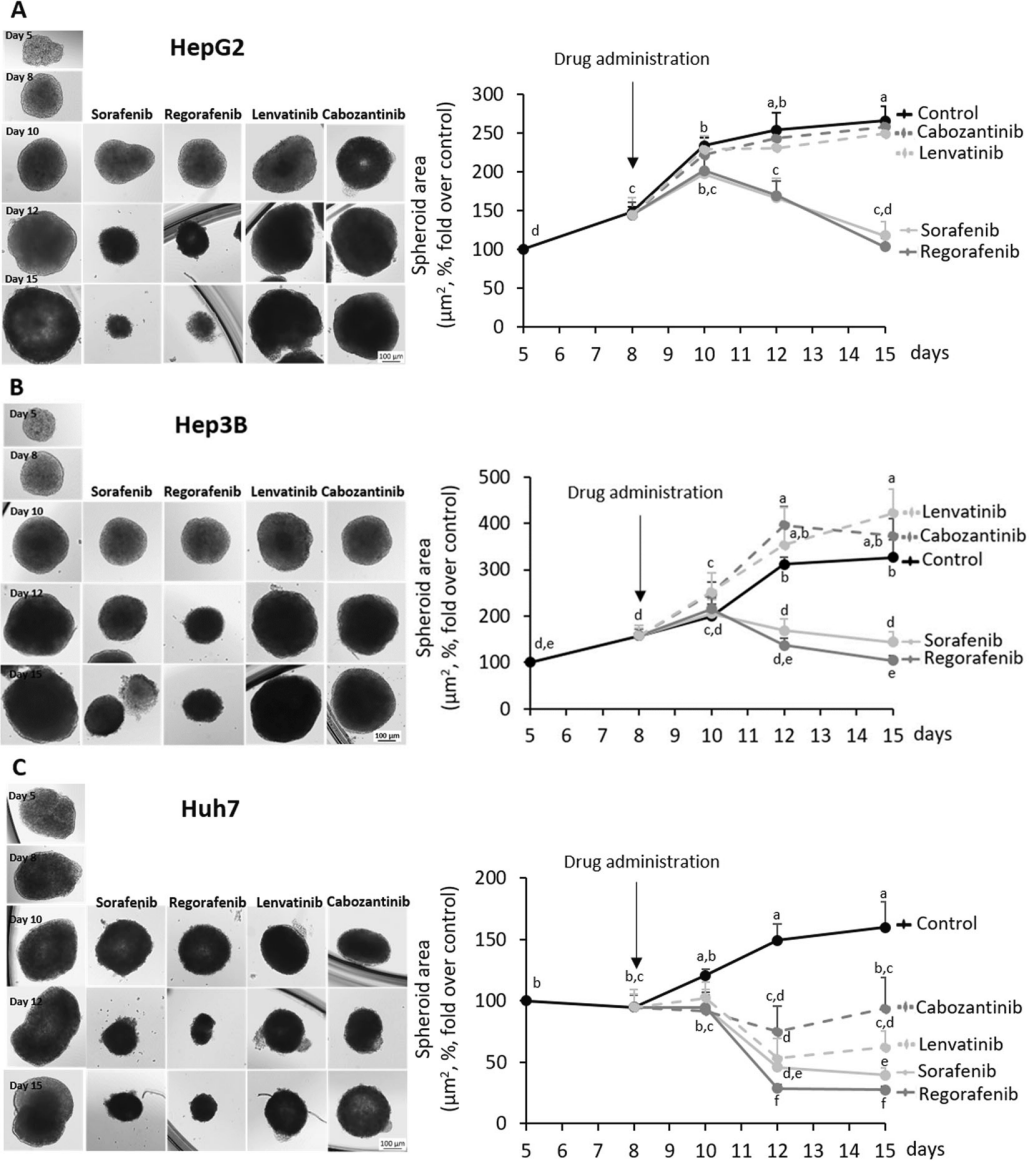
Drug effectiveness in liver cancer cells cultured in spheroids. Effect of Sorafenib, Regorafenib, Lenvatinib, and Cabozantinib in the area of spheroids generated by HepG2 (**a**), Hep3B (**b**), and Huh7 (**c**) cells. Drugs (10µM) were administered at day 8th after spheroid establishment, and cultures were maintained up to day 15th as described in “Materials and methods” section. The area of the spheroids (µm^2^, %, fold over control) were measured at days 8th, 10th, 12th, and 15th. All results are expressed as mean±SD of independent experiments (*n* = 3). The groups with statistically significant differences among them (*p* ≤ 0.05) were indicated with different letters (a, b, c, d, e, or f). Magnification of images are ×10.

**Fig. 2:**
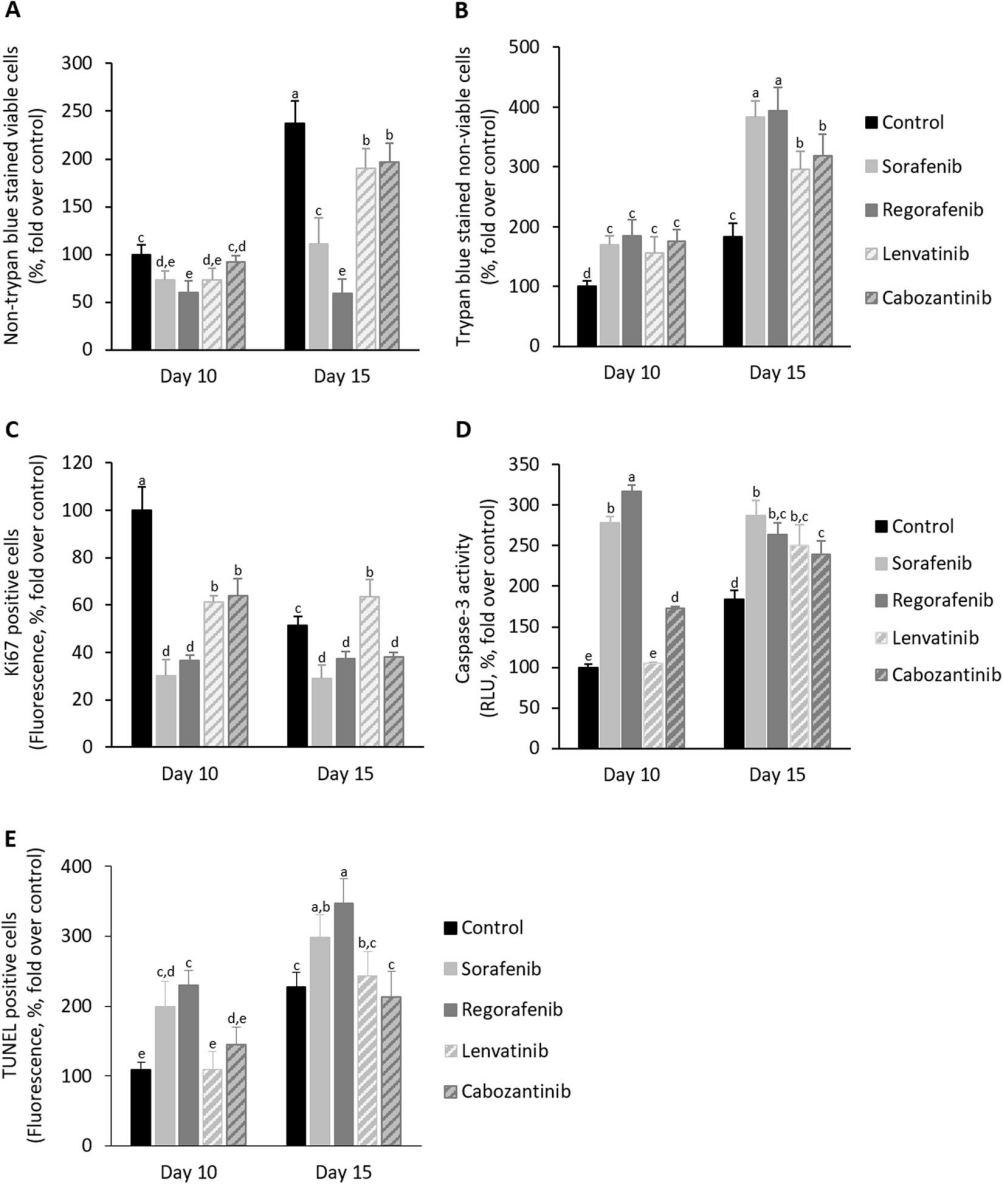
Drug effectiveness in HepG2 cells cultured in spheroids. Effect of Sorafenib, Regorafenib, Lenvatinib, and Cabozantinib in non-trypan blue-stained viable cells **(a)**, trypan blue non-viable cells (**b**), Ki67-positive cells (**c**), caspase-3 activity (**d**), and TUNEL-positive cells (**e**) in spheroids generated by HepG2 cells. Drugs (10µM) were administered at day 8th after spheroid establishment, and cultures were maintained up to day 15th as described in “Material and methods” section. The parameters were measured at days 10th and 15th. Trypan blue staining in cells from trypsin-dissociated spheroids allowed the identification of viable and non-viable cells (%, fold over control). Ki67- and TUNEL-positive cells were determined by immunohistochemistry, and caspase-3 activity was assessed using commercial caspase-Glo® Assay Systems as described in “Materials and methods” section. Ki67- and TUNEL-positive cells were assessed by fluorescence methods (fluorescence, %, fold over control). Caspase-3 activity is shown as the RLU (%, fold over control). All results are expressed as mean±SD of independent experiments (*n* = 3). The groups with statistically significant differences among them (*p* ≤ 0.05) were indicated with different letters (a, b, c, d, or e).

**Fig. 3:**
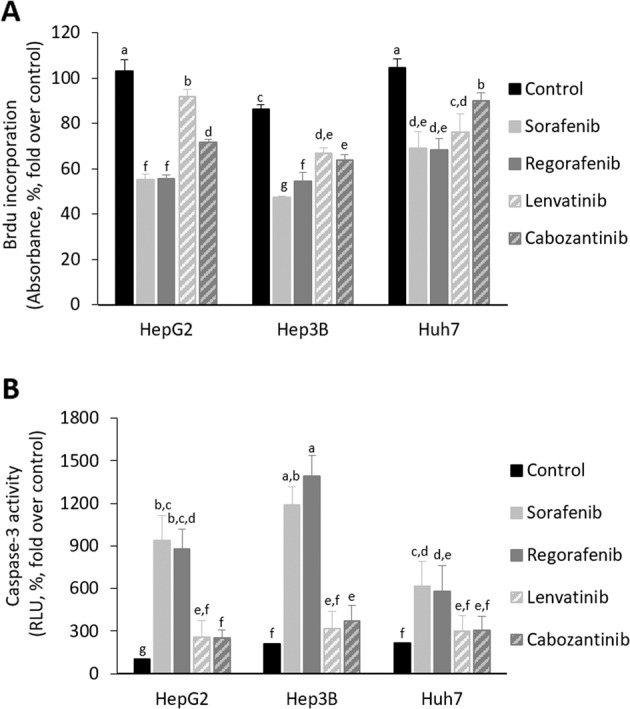
Drug effectiveness in liver cancer cells cultured in monolayer. Effect of Sorafenib, Regorafenib, Lenvatinib, and Cabozantinib in BrdU incorporation (**a**) and caspase-3 activity (**b**) in HepG2, Hep3b, and Huh7 cells cultured in 2D system. Drugs (10µM) were administered at 24h after plating. BrdU incorporation and caspase-3 activity were determined 24h after drug administration using commercial colorimetric assay and caspase-Glo® Assay Systems as described in “Materials and methods” section, respectively. BrdU incorporation is shown as the absorbance at 370nm (reference wavelength: 492nm; absorbance, %, fold over control). Caspase-3 activity is shown as the RLU (%, fold over control). All results are expressed as mean ± SD of independent experiments (*n* = 4). The groups with statistically significant differences among them (*p* ≤ 0.05) were indicated with different letters (a, b, c, d, e, f, or g).

The administration of Sorafenib and Regorafenib strongly reduced the area of spheroids generated from HepG2, Hep3B, and Huh7 cells (Fig. 1a–c, Supplementary Table 1). Lenvatinib and Cabozantinib appeared to be effective in Huh7 (Fig. 1c, Supplementary Table 1), but not in HepG2 and Hep3B cell lines (Fig. 1a, b, Supplementary Table 1). Sorafenib and Regorafenib reduced Ki67-positive cells (Fig. 2c), as well as increased caspase-3 activity (Fig. 2d) and TUNEL-positive cells (Fig. 2e) at day 10th, and while reduced non-trypan blue-stained viable cells (Fig. 2a) and increased trypan blue-stained non-viable cells (Fig. 2b) at day 15th in spheroids more strongly than Lenvatinib and Cabozantinib in cultured spheroids. The increased antiproliferative and proapoptotic effectiveness of Sorafenib and Regorafenib versus Lenvatinib and Cabozantinib (10 µM) in spheroids was further assessed in 2D cultured HepG2, Hep3B, and Huh7 cells (24 h, Fig. 3). BrdU incorporation (Fig. 3a) and caspase-3 activity (Fig. 3b) in 2D cultured HepG2, Hep3B, and Huh7 cell lines partially confirmed 3D data. Sorafenib and Regorafenib exerted potent antiproliferative and proapoptotic effects in decreasing order of effectiveness in HepG2 ≥ Hep3B ≥ Huh7 cultured in 2D system (Fig. 3a, b). Lenvatinib and Cabozantinib were also able to reduce cell proliferation (Fig. 3a), and at low extend increased caspase-3 activity in HepG2 cells (Fig. 3b), in HCC cells cultured in monolayer.

### Dose–response antiproliferative and proapoptotic properties of Sorafenib, Regorafenib, Lenvatinib, and Cabozantinib in primary human hepatocytes and HCC cell lines according to differentiation and p53 status

**Fig. 4:**
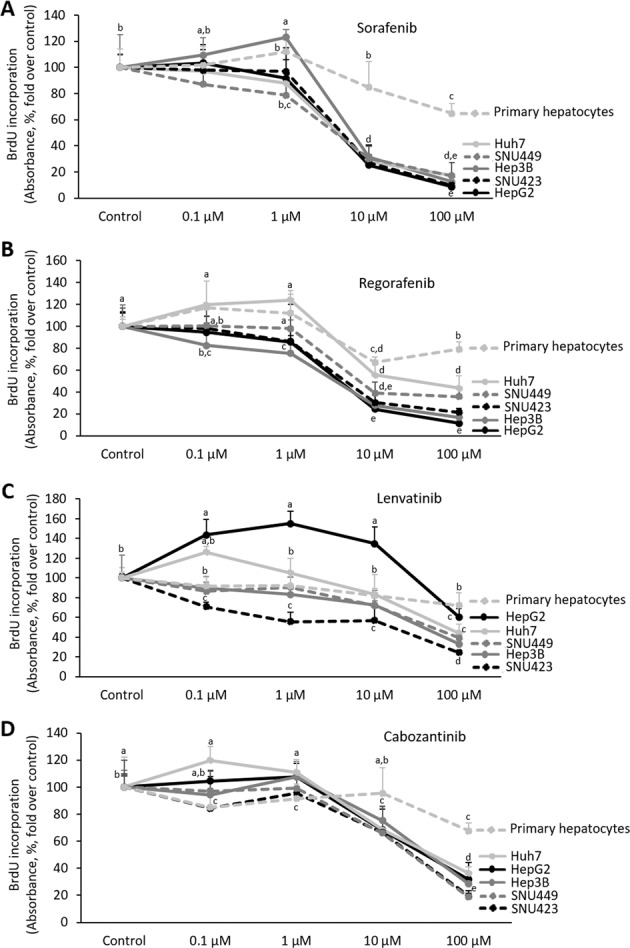
Drug effectiveness on cell proliferation in liver cancer cells cultured in monolayer. Effect of Sorafenib (**a**), Regorafenib (**b**), Lenvatinib (**c**), and Cabozantinib (**d**) in BrdU incorporation in HepG2, Hep3b, Huh7, SNU423, SNU449, and primary human hepatocytes cultured in 2D system. Graphs are separated according to treatments. Drugs (0, 100nM, 1µM, 10µM, and 100µM) were administered at 24h after plating. BrdU was determined 24h after drug administration using a commercial colorimetric assay, as described in as described in “Materials and methods” section. BrdU incorporation is shown as the absorbance at 370nm (reference wavelength: 492nm; absorbance, %, fold over control). All results are expressed as mean±SD of independent experiments (*n* = 6). The groups with statistically significant differences among them (*p* ≤ 0.05) were indicated with different letters (a, b, c, d, or e).

Sorafenib (10 µM) exerted a potent antiproliferative activity at 24 h in all HCC cell lines (Fig. 4a). Regorafenib (10 µM) also exerted, but at lower extent than Sorafenib, antiproliferative activity in decreasing order HepG2 ≥ Hep3B ≥ SNU423 ≥ SNU449 ≥ Huh7. However, Regorafenib at the recommended dose (1 µM) induced a moderate reduction of cell proliferation in HepG2 and Hep3B compared with untreated cells (Fig. 4b). Lenvatinib and Cabozantinib appeared to be more active in SNU423, SNU449, and Hep3B cells than HepG2 and Huh7 (Fig. 4c, d, respectively). In particular, Lenvatinib (0.1 µM) and Cabozantinib (1 µM) reduced cell proliferation in SNU423, as well as SNU423 and SNU449, cell lines (Fig. 4c, d, respectively).

**Fig. 5:**
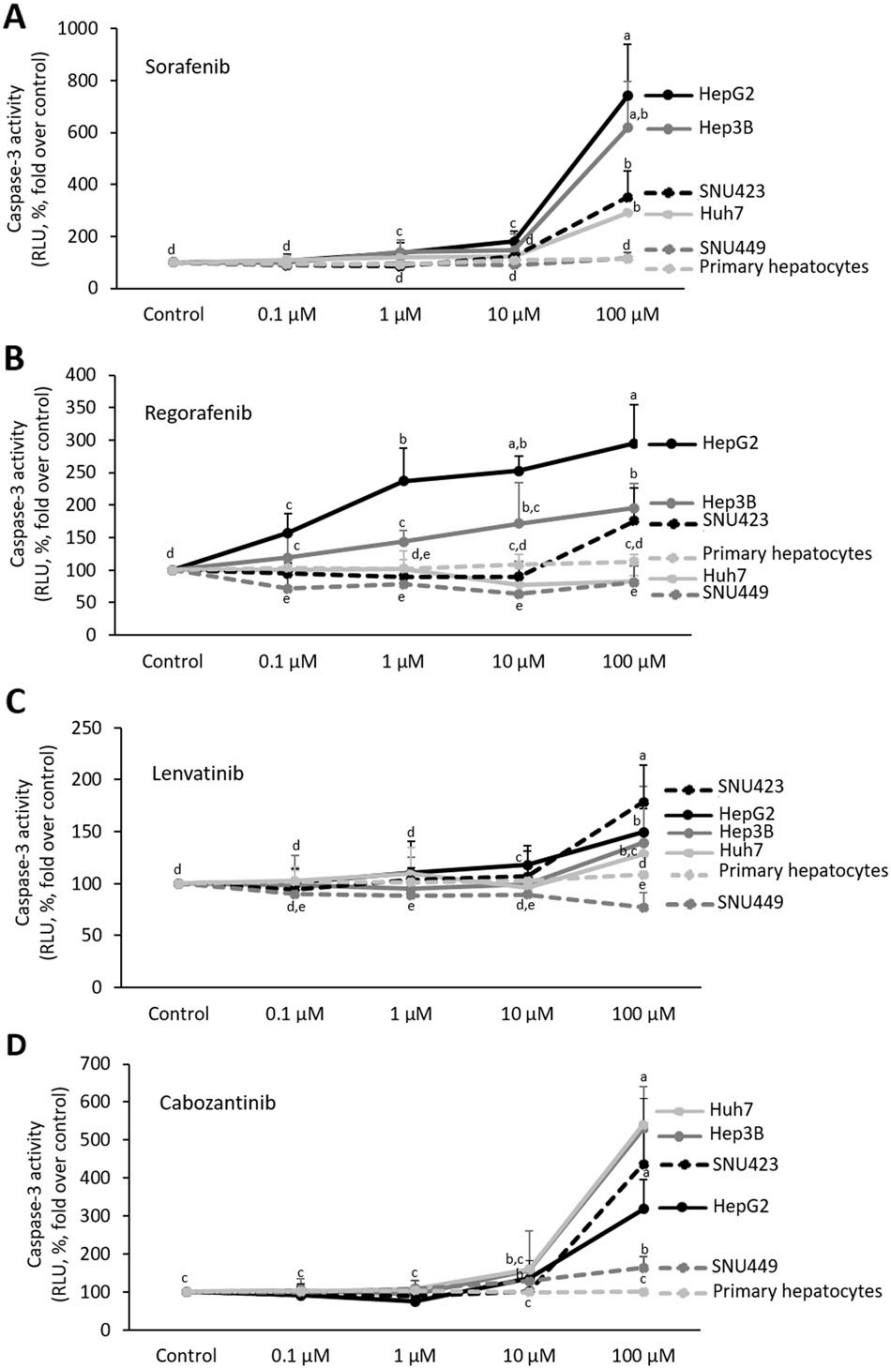
Drug effectiveness on apoptosis in liver cancer cells cultured in monolayer. Effect of Sorafenib (**a**), Regorafenib (**b**), Lenvatinib (**c**), and Cabozantinib (**d**) in caspase-3 activity in HepG2, Hep3b, Huh7, SNU423, SNU449, and primary human hepatocytes cultured in 2D system. Graphs are separated according to treatments. Drugs (0, 100nM, 1µM, 10µM, and 100µM) were administered at 24h after plating. Caspase-3 activity was determined 24h after drug administration using a commercial caspase-Glo® Assay Systems as described in “Materials and methods” section. Caspase-3 activity is shown as the RLU (%, fold over control). All results are expressed as mean±SD of independent experiments (*n* = 6). The groups with statistically significant differences among them (*p* ≤ 0.05) were indicated with different letters (a, b, c, d, or e).

Sorafenib (10 µM) induced caspase-3 activity in HepG2 and Hep3B (Fig. 5a). The highest dose of Sorafenib (100 µM) induced a significant rise of the downstream caspase activity in HepG2, Hep3B, SNU423, and Huh7 cells. Regorafenib (10 µM) also exerted, but at lower extent than Sorafenib, proapoptotic activity in decreasing order HepG2 ≥ Hep3B ≥ SNU423 ≥ Huh7 ≥ SNU449. Regorafenib (1 µM) induced caspase-3 activity in HepG2 and Hep3B compared with untreated cells (Fig. 5b). Although the dose–response study showed that the highest dose (100 µM) of Lenvatinib and Cabozantinib increased caspase-3 activity at variable extend in all cell lines; however, Lenvatinib (0,1 µM) and Cabozantinib (1 µM) were not able to induce caspase-3 in any cell lines tested (Fig. 5c, d, respectively).

### Alteration of mitochondrial respiration in HCC cells

We have assessed OCR, mitochondrial-dependent ATP generation, and maximal respiration capacity at basal conditions in all cell lines. HepG2, Huh7, and Hep3B showed an increase in basal OCR and ATP generation than SNU423 and SNU449 cell lines (Table 1). HepG2 and Huh7, but not Hep3B, had higher maximal respiration capacity than SNU423 and SNU449 (Table 1).

**Table 1:** Basal oxygen consumption rate (OCR), mitochondrial-dependent ATP generation, and maximal respiration capacity of HepG2, Huh7, Hep3B, SNU423, and SNU449 cell lines cultured in 2D system.

Cell lines	OCR	ATP generation	Maximal respiration capacity
HepG2	5.73 ± 0.429^a^	3.86 ± 1.353^a^	12.52 ± 0.801^b^
Huh7	7.04 ± 0.728^a^	3.45 ± 1.412^a^	20.26 ± 1.312^a^
Hep3B	5.50 ± 0.442^a^	4.52 ± 0.624^a^	10.52 ± 0.962^b,c^
SNU423	1.97 ± 0.366^b^	1.43 ± 0.213^b^	2.43 ± 0.620^d^
SNU449	3.46 ± 1.061^b^	0.45 ± 0.263^c^	9.18 ± 1.103^c^

The parameters were measured in cells 48 h after plating and expressed (pmol/min/µg protein) according to MitoStress kit protocol in a XFp Extracellular Flux Analyzer as described in “Materials and methods” section. All results are expressed as mean ± SD of independent experiments (*n* = 6). The groups with statistically significant differences (*p* ≤ 0.05) were indicated with different letters (a, b, c, or d).

### Expression of tyrosine kinase receptor upon treatments in HCC cells

**Fig. 6:**
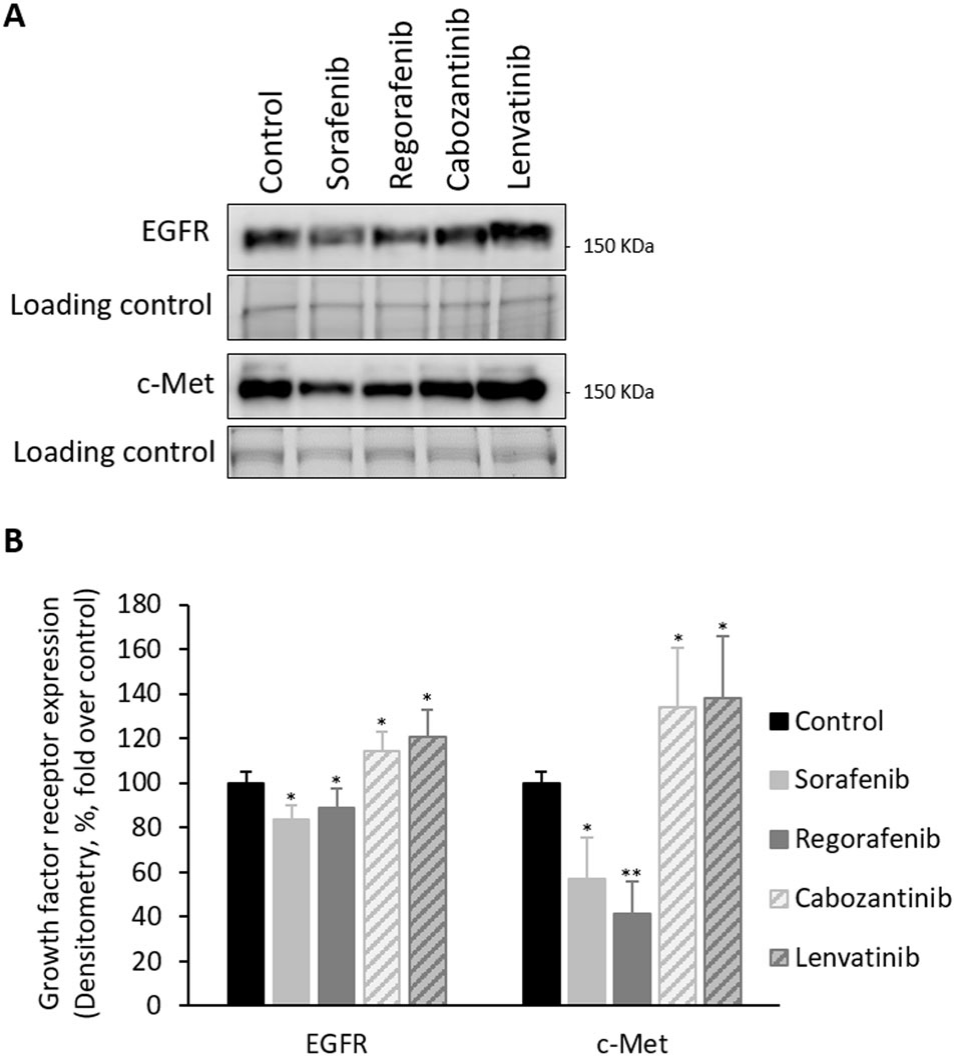
Drug effectiveness on cell proliferation receptor protein expression in HepG2 cells cultured in monolayer. Effect of Sorafenib, Regorafenib, Lenvatinib, and Cabozantinib in the protein expression of EGFR and c-Met in HepG2 cells cultured in 2D system. Drugs (10µM) were administered at 24h after plating. The expression of tyrosine kinase receptors was assessed 24h after drug administration by SDS–PAGE electrophoresis coupled to western blot procedure as described in “Materials and methods” section. The values were obtained by densitometric analysis of the spots in relation to their loading control in the blots (densitometry, %, fold over control). All results are expressed as mean±SD of independent experiments (*n* = 3). The level of significance was set at *p ≤ 0.05 and **p ≤ 0.01 in comparison with their corresponding control.

The administration of regular used in vitro dose (10 µM) of Sorafenib and Regorafenib reduced EGFR and c-Met protein expressions, while Lenvatinib and Cabozantinib increased EGFR and c-Met protein expressions (Fig. 6). The protein expression of VEGFR, PDGR, and FGFR was not altered by the treatments (data are not shown).

## Discussion

Two-thirds of patients with HCC are diagnosed in advanced stages receiving treatments as first- (Sorafenib and Lenvatinib) or second- (Regorafenib and Cabozantinib) line therapies^2–6^. However, drug resistance is widely observed and only a minor percentage of patients are effectively extending their survival after treatment^21^.

Sorafenib and Regorafenib exerted a more potent relative beneficial properties than Lenvatinib and Cabozantinib in reducing spheroid area (Fig. 1, Supplementary Table 1), cell proliferation (Fig. 2a, c, as well as Fig. 3), and promoting cell death (Fig. 2b, d, e, as well as Fig. 3b) in 3D and 2D cultured-differentiated HCC cells. However, a detailed analysis showed differences in drug response in 3D and 2D cell culture models. The reduction of spheroid area by Sorafenib and Regorafenib was similar in all cell lines cultured in spheroids (Fig. 1, Supplementary Table 1); however, the increase of caspase-3 activity, and at lower extent the reduction of BrdU incorporation, showed a significant decreasing order of effectiveness of the drugs in HepG2 ≥ Hep3B ≥ Huh7 cells cultured in monolayer (Fig. 3). Lenvatinib and Cabozantinib that reduced spheroid area in 3D cultured Huh7, but not in HepG2 and Hep3B (Fig. 1, Supplementary Table 1), appeared to reduce BrdU incorporation (Fig. 3a), and at low extent to increase caspase-3 activity (Fig. 3b), in all 2D cultured cell lines. These data might suggest that although a parallelism existed in drug response in 3D and 2D models, it can be feasible that the increased hypoxic environment in spheroids, which does not occur in cells cultured in monolayer, was inducing cell resistance to the antiproliferative and proapoptotic properties induced by the treatments. In fact, the enhanced expression of HIF-2alpha isoform causes a survival advantage in HepG2 cells cultured in spheroids^22^.

An increase in non-trypan blue-stained viable cells (Fig. 2a), trypan blue-stained non-viable cells (Fig. 2b), caspase-3 activity (Fig. 2d), and TUNEL-positive cells (Fig. 2e), and reduction in Ki67-positive cells (Fig. 2c) at day 10th were observed compared to day 15th in spheroids from control HepG2 cells. These changes suggested the existence of active cell proliferation at day 10th that progressed, but also coexisted with cell cycle arrest and apoptosis, at day 15th in spheroids. The presence of released autocrine factors or physical constraint might occur in our experimental conditions at day 15th after spheroid establishment. In addition, the strong reduction of Ki67-positive cells (Fig. 2c), and the increase of caspase-3 activity (Fig. 2d) and TUNEL-positive cells (Fig. 2e) induced by Sorafenib and Regorafenib at day 10th were associated with the reduction of non-trypan blue-stained viable cells (Fig. 2a), and the increase of trypan blue-stained non-viable cells (Fig. 2b) at day 15th in HepG2. This situation showed that the early activation of cell death and blockage of cell proliferation by Sorafenib and Regorafenib observed at day 10th had an impact in the number of viable and dead cells at day 15th in spheroids.

p53, a tumor suppressor gene involved in cell cycle control, DNA repair, apoptosis, and cell differentiation, is one of the most mutated genes (up to 67% according to the different studies) in HCC although mutation prevalence varies greatly depending on etiology^23^. The expression of p53 and other p53-related components of the gene family are transcriptionally regulating the expression of cell death receptors and apoptosis^24,25^. An association appears between p53 mutations, and the degree of cell dedifferentiation and survival in patients with HCC^26,27^. The experiments suggested that Sorafenib and Regorafenib compared with Lenvatininb and Cabozantinib were especially effective in HepG2 with wild-type 53, while a relative decreasing antiproliferative and proapoptotic properties were observed in 2D cultured Hep3B and Huh7 cell lines (Fig. 3a, b, respectively). This pattern of action is in agreement with our previous study that showed a more potent proapoptotic and antiproliferative effects of Sorafenib in HepG2 cells than Hep3b and Huh7 cells^10^. Sorafenib has also been shown to upregulated p53 and promotes p53-dependent apoptosis^9,28^. The lack of functional p53 in most HCC cells have been proposed as a possible mechanism for Sorafenib resistance^28,29^. The overexpression of p53 renders Hep3B more sensitive to Sorafenib, while p53 knockdown from HepG2 cells increased their resistance^30^.

The clinical trials have established recommended dose for Sorafenib (800 mg/24 h), Regorafenib (160 mg/24 h), Lenvatinib (<60 kg: 8 mg/24 h; ≥60 kg: 12 mg/24 h), and Cabozantinib (60 mg/24 h) in the treatment of patients in advanced stage of HCC. The dose–response analysis enclosed concentrations found in blood from advanced HCC-treated patients. Sorafenib (10 µM), and at lower extent Regorafenib (1 µM), exerted the strongest effects in cultured HepG2 cell line, while the response was lower in 2D cultured Huh7, SNU423, and SNU449 (Figs. 4 and 5). Although, Lenvatinib and Cabozantinib showed to exert moderate antiproliferative effects at their recommended dose in SNU423, and SNU423 and SNU449, respectively (Fig. 4c, d, respectively). However, our study also showed that Lenvatinib and Cabozanitib exerted increased activity at higher dose than those recommended (0.1 µM and 1 µM, respectively; Figs. 4 and 5).

The induction of cell death by Sorafenib and Regorafenib has been related to mitochondrial dysfunction in cultured hepatocytes and liver mitochondria fraction from rats^31,32^. We have observed that basal OCR and mitochondrial-dependent ATP generation of SNU423 and SNU449 were significantly lower than observed in HepG2, Huh7, and Hep3B (Table 1). In addition, HepG2 and Huh7, but not Hep3B cells, had higher maximal respiration capacity than SNU423 and SNU449 (Table 1). Hep3B and SNU449, which show increased number of mesenchymal phenotype markers than PLC/PRF/5 and Huh7, are associated with an increased expression of transforming growth factor β (TGF-β) and vimentin, as well as shunt glucose-6-phosphate to the pentose phosphate pathway (PPP), overall parallel with the reduction in E-cadherin expression and OCR^33^. The proapoptotic effectiveness of Sorafenib are associated with mitochondrial dysfunction and reduction of glycolysis in HepG2 cells^19^. In this study, the lack of effectiveness of low dose of Sorafenib (10 nM) compared with the recommended dose (10 µM) was associated with an increased mitochondrial function and glycolysis^19^. In concordance, Fiume et al.^34^ showed that the reduced decline of cell viability in SNU449 compared with that observed in PLC/PRF/5 by Sorafenib (4 and 8 µM) was associated with resistance to mitochondrial respiration downregulation. All this data might suggest that Lenvatinib and Cabozantinib, differently to Sorafenib and Regorafenib, have a relative higher antitumoral activity in cells with reduced mitochondrial respiration, increased glycolytic and PPP pathways that are associated with increased epithelial–mesenchymal transition (EMT) phenotype.

The resistance of HCC cells during Sorafenib administration has been related to the upregulation of survival cell signaling mediated through insulin growth factor receptor (IGFR)^35,36^, c-Met^37^, and FGFR^36^. c-Met phosphorylation and activation of mTOR are also related to resistance to Sorafenib in patient-derived HCC xenograft^37^. Tovar el al.^36^ have shown Sorafenib (5 µM) reduces cell viability in Hep3B (55%) and Huh6 (29%). Huh6 cell line, which express wild-type p53, shows increased number of markers and morphology features of undifferentiated cells than Hep3B cell line^38^. The resistance of Hep3B and Huh6 to Sorafenib is associated with FGFR^36^, and EGFR^39^, or FGFR^36^ upregulation, respectively. Linsitinib (5 µM), a dual TKI of IGF1R/IR, and the pan-FGFR inhibitor BGJ398, increased the effectiveness of Sorafenib in both resistant cell lines^36^. The increased effectiveness of Sorafenib and Regorafenib (Figs. 3–5), in comparison with Lenvatinib and Cabozantinib, was related to the downregulation of EGFR and c-Met protein expression (Fig. 6) in 2D cultured HepG2 cells.

Fernando et al.^9^ have shown that Sorafenib sensitizes HCC cells to the apoptotic activity of TGF-β through the intrinsic pathway and tumor necrosis factor-α (TNF-α)-dependent extrinsic pathway. The proapoptotic activity of Sorafenib is associated with downregulation of S-nitrosylation in cell death receptors in liver cancer cells^40^. In concordance with our study, untransformed hepatocytes did not respond to Sorafenib-induced cell death^9^. Sorafenib, Regorafenib, Lenvatinib, and Cabozantinib exerted a minor effect in cell proliferation and death in primary human hepatocytes (Figs. 4 and 5).

Different reports have suggested an association between EMT and chemoresistance in liver tumor cells. SNU449, HLF, and HLE liver cancer cell lines that express mesenchymal markers (CD44, vimentin, and snail) are refractory to Sorafenib treatment compared to HepG2, Hep3B, PLC/PRF/5-expressing epithelial markers (E-cadherin and CK-18)^41,42^. Similar conclusions were obtained by Van Zijl et al.^43^ using HCC cells derived from patients. In fact, factors turning back mesenchymal to epithelial phenotypes increase responsiveness of HCC cells to Sorafenib^44^. The expression of EMT markers appeared to be more relevant than upregulation of EGFR expression or downstream activation of ERK signaling for the sensitivity of tumor cells to EGFR inhibitors^45^. The EMT status also predicts HCC cell sensitivity to IGFR inhibitors in HCC cells^46^. A recent gene expression classification of HCC has identified a poor survival subclass termed S2 that express E-cadherin, c-myc, and FGFR3-4 protein expression^47^. Intriguingly, S2 gene signature that included Hep3B, HepG2, and HuH7, but not SNU423, cell lines that were highly susceptible to inhibition of cell proliferation by FGFR1-4 inhibitors^47^. In our conditions, Lenvatinib that targets FGFR3/4, showed lower antiproliferative and proapoptotic activities in HepG2, Hep3B, and HuH7 than SNU423 cell lines (Figs. 4–5).

In conclusion, the present study showed that although a parallelism existed in the effectiveness of drugs in well-differentiated cells cultured in 3D and 2D models, it might be that the hypoxic environment generated in spheroids would be responsible for the minor effectiveness of drugs in cells cultured in the 3D system in comparison with that observed in 2D cultured cells. The administration of regular used in vitro dose (10 µM) in 3D and 2D cultures, as well as the dose–response analysis in 2D cultures showed that Sorafenib and Regorafenib were increasingly effective reducing cell proliferation, and inducing apoptosis in well-differentiated and wild-type p53 HCC cells. Lenvatinib and Cabozantinib were more effective than Sorafenib and Regorafenib in moderately to poorly differentiated cells with mutated or lacking p53 HCC cells. The study also suggested that the highest effectiveness of Sorafenib and Regorafenib might be associated with high mitochondrial respiration, in comparison with Lenvatinib and Cabozantinib that appeared to be more active in cells with low basal OCR, mitochondrial-dependent ATP generation, and maximal respiration capacity.

### Supplementary information

Supplementary Figure 1

Supplementary Table 1

Supplementary figure and table legends
