# Correction to: Differential effectiveness of tyrosine kinase inhibitors in 2D/3D culture according to cell differentiation, p53 status and mitochondrial respiration in liver cancer cells

**DOI:** 10.1038/s41419-024-06953-7

**Published:** 2024-08-20

**Authors:** María A. Rodríguez-Hernández, Raquel Chapresto-Garzón, Miryam Cadenas, Elena Navarro-Villarán, María Negrete, Miguel A. Gómez-Bravo, Victor M. Victor, Francisco J. Padillo, Jordi Muntané

**Affiliations:** 1 Institute of Biomedicine of Seville (IBiS), Hospital University “Virgen del Rocío”/CSIC/University of Seville, Seville, Spain; 2 Spanish Network for Biomedical Research in Hepatic and Digestive diseases (CIBERehd), Institute of Health Carlos III (ISCIII), Madrid, Spain; 3 Department of General Surgery, Hospital University “Virgen del Rocío”/CSIC/University of Seville/IBIS, Seville, Spain; 4 Service of Endocrinology, University Hospital Doctor Peset, Foundation for the Promotion of Health and Biomedical Research in the Valencian Region (FISABIO), Valencia, Spain; 5 Department of Physiology, University of Valencia, Valencia, Spain

Correction to: *Cell Death and Disease* 10.1038/s41419-020-2558-1, published online 07 May 2020

In this article, the spheroid image obtained after 15 days of treatment of Hep3B with Cabozantinib was incorrectly duplicated from that obtained after 12 days. The new Fig. 1B shows the correct spheroid image at 15 days. The replacement does not change the results or conclusions of Fig. 1B. The authors apologize for the unexpected error in the preparation of Fig. 1B.

Original Fig. 1B
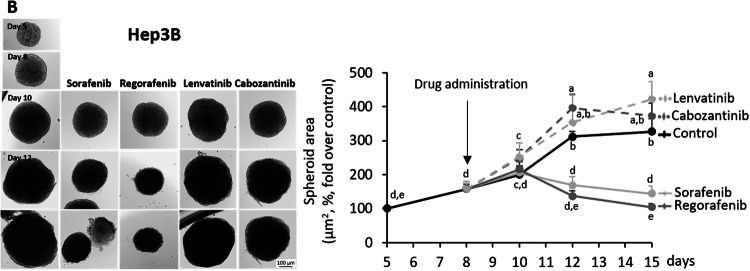


Corrected Fig. 1B
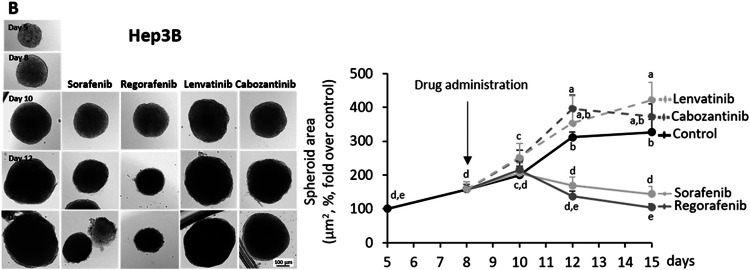


The original article has been corrected.

