# Supplementary figures and images for: Differential effectiveness of tyrosine kinase inhibitors in 2D/3D culture according to cell differentiation, p53 status and mitochondrial respiration in liver cancer cells
Source: Cell Death Dis. 2020 May 7;11(5):339. doi: 10.1038/s41419-020-2558-1 (PMC7206079; doi:10.1038/s41419-020-2558-1)

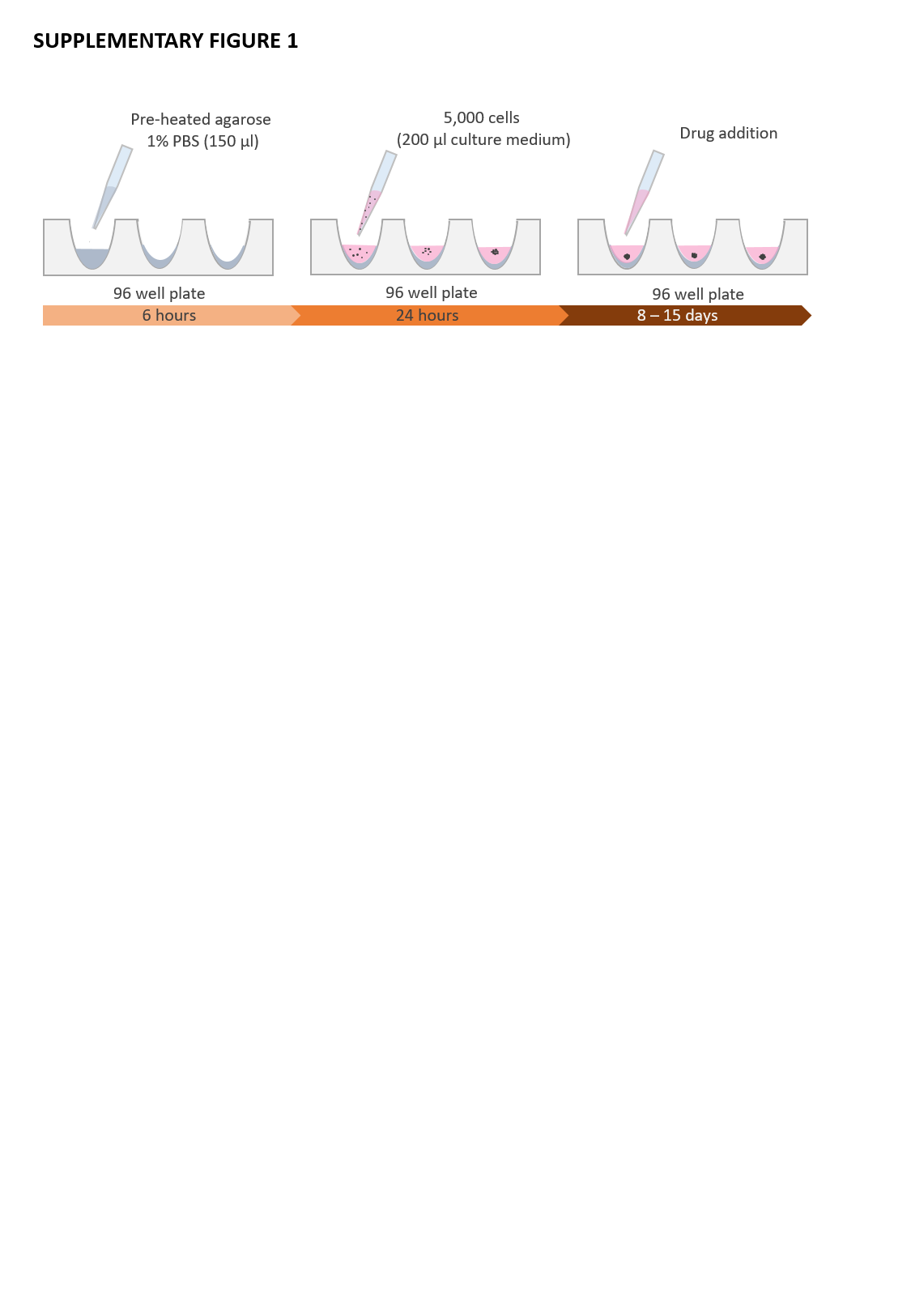

Supplement: Supplementary file 1 — Supplementary Figure 1 [file 41419_2020_2558_MOESM1_ESM.tif]
