# Supplementary material for: Differential effectiveness of tyrosine kinase inhibitors in 2D/3D culture according to cell differentiation, p53 status and mitochondrial respiration in liver cancer cells
Source: Cell Death Dis. 2020 May 7;11(5):339. doi: 10.1038/s41419-020-2558-1 (PMC7206079; doi:10.1038/s41419-020-2558-1)
Supplement: Supplementary file 2 — Supplementary Table 1 [file 41419_2020_2558_MOESM2_ESM.docx]

|  | **HepG2**  **(%, fold over control)** | **Hep3B**  **(%, fold over control)** | **Huh7**  **(%, fold over control)** |
| --- | --- | --- | --- |
| **Control** | 98138 ± 4836^a^ (100 %) | 97067 ± 2331^b^ (100 %) | 119875 ± 22146^a^ (100 %) |
| **Sorafenib** | 43476 ± 2555^c,d^ (44 %) | 42042 ± 5425^d^ (43 %) | 28824 ± 3898^e^ (24 %) |
| **Regorafenib** | 38055 ± 3296^c,d^ (39 %) | 30364 ± 6982^e^ (31 %) | 18776 ± 3981^f^ (15 %) |
| **Lenvatinib** | 92071 ± 2995^a^ (94 %) | 127334 ± 20674^a^ (131 %) | 46153 ± 7294^c,d^ (38 %) |
| **Cabozantinib** | 95480 ± 5340^a^ (97 %) | 109182 ± 17815^a,b^ (112 %) | 65874 ± 16383^b,c^ (55 %) |
