# Supplementary material for: Differential effectiveness of tyrosine kinase inhibitors in 2D/3D culture according to cell differentiation, p53 status and mitochondrial respiration in liver cancer cells
Source: Cell Death Dis. 2020 May 7;11(5):339. doi: 10.1038/s41419-020-2558-1 (PMC7206079; doi:10.1038/s41419-020-2558-1)
Supplement: Supplementary file 3 — Supplementary figure and table legends [file 41419_2020_2558_MOESM3_ESM.docx]

**Supplementary Figure 1.** Time-related procedure for multicellular spheroid generation according to the liquid overlay technique in 1 % agarose-coated 96-well plates as described in Material and Methods.

**SUPPLEMENTARY TABLE 1.** Area of spheroids generated from HepG2, Hep3B and Huh7 cells treated with Sorafenib, Regorafenib, Lenvatinib and Cabozantinib. Spheroids were obtained according to the procedure described in Material and Methods. Drugs (10 µM) were administered at 8^th^ days after spheroid establishment. The indicated spheroid areas (µm^2^) were measured at 15^th^ days after spheroid establishment. Data are expressed as mean ± SD of independent experiments (n=3). The groups with statistically significant differences (p≤0.05) were indicated with different letters (a, b, c, d, e or f).
